# The reproductive success of bovine sperm after sex-sorting: a meta-analysis

**DOI:** 10.1038/s41598-021-96834-2

**Published:** 2021-08-30

**Authors:** Sven Reese, Miguel Camara Pirez, Heather Steele, Sabine Kölle

**Affiliations:** 1 School of Veterinary Medicine, Institute of Veterinary Anatomy, Histology and Embryology, LMU Munich, Munich, Germany; 2 School of Medicine, Health Sciences Centre, University College Dublin (UCD), Dublin, Ireland

**Keywords:** Biotechnology, Developmental biology

## Abstract

In the three decades since its inception, the sex-sorting technology has progressed significantly. However, field studies report conflicting findings regarding reproductive outcomes. Therefore, we conducted this meta-analysis of all trials published between 1999 and 2021. Non-return rates after 24 or 60 d (NRR 24/60), pregnancy, calving, abortion, and stillbirth rates were compared after AI with sex-sorted vs non-sorted sperm. Additionally, the impact of recent developments in the sex-sorting technology was assessed. Of 860 studies found, 45 studies (72 trials) provided extractable data and were included. Overall, the results of this meta-analysis provided evidence that the NRR 24/60 was diminished by 13%, pregnancy rates were reduced by 23% (25% cows, 21% heifers) and calving rates were reduced by 24% when using sex-sorted sperm. Enhancing the dosage to 4 million sex-sorted sperm/straw (including recent improvements, high vs low dose) as well as using fresh sex-sorted sperm (sorted vs non-sorted) increased pregnancy rate ratios by 7 percentage points. The refinement of the sex-sorting technology after 2015 resulted in a lowered reduction of pregnancy and calving rate of 19% and 23%, respectively. Whereas abortion rates were similar, the stillbirth of male calves was increased by 6.3%.

## Introduction

Sex-sorting of bovine spermatozoa was established through the development of flow cytometric sorting in the late 1980s with the first live calf being born in 1993^1^. In the three decades since the development of sex-sorted sperm, its use has been integrated into many farming systems globally. As both the dairy and beef industries face mounting pressure to increase farm efficiency with less available agricultural land^2^, the potential for greater integration across all farming systems of sex-sorted sperm for AI is promising. Furthermore, the increased focus on animal welfare highlights the necessity to reduce the surplus of male calves in the dairy industry^3^.

The technology for sex-sorting sperm was developed at the Lawrence Livermore National Laboratory (LLNL, CA) which established the technology for orientation of the sperm enabling precise DNA content recordings^4^. In collaboration with USDA, Oklahoma State University, and LLNL the technology was further developed to precisely determine the DNA content differences between X and Y bearing sperm for cattle, pigs, sheep and rabbits^5^. Maintaining the viability of sperm was achieved by labelling with the dye Hoechst 33342 instead of DAPI^5,6^.

The combination of this labelling method and the sorting technology from LLNL at the USDA Beltsville Agricultural research centre led to the establishment of an early sex-sorting protocol. A major breakthrough was the first reported live offspring born using this technology in rabbits in 1989^7^. In 1991, the methodology was patented^8^. Initially, flow cytometry limitations could only accurately resolve 350,000 sperm/hr so that standard insemination doses of bovine cryopreserved sperm of 20 × 10^6^ were not achievable^7^. When field trials revealed that insemination doses ranging from 1 × 10^6^ to 2.5 × 10^6^ sperm achieved sufficient conception rates^9,10^ the USDA granted a license to the Colorado State University Research Foundation, under the company XY Inc., to begin the commercialisation of this technology named Beltsville Sperm Sexing Technology. XY Inc. was acquired by Sexing Technologies (Navasota, TX, USA) in 2007^11^.

In this sorting process sperm DNA is stained stoichiometrically with Hoechst 33342 before being pumped in a stream passing a laser at specific wavelengths^12^. The Hoechst 33342 stained sperm emit a bright blue fluorescence when illuminated, which is measured by a photomultiplier^13^. Using a crystal vibrator sperm are forced into individual droplets. Opposite charges are applied to droplets containing X or Y bearing sperm. The droplets then pass electrical fields which forces them into streams for collection. Droplets which remain uncharged due to inadequate sperm orientation or sperm death are discarded.

In the following decades, the technology underwent further improvements. As the low sorting throughput rate was a main barrier to commercial success, the flow cytometric system, the MoFlo™ cytometer, first underwent modifications to its nozzle so that an increased number of sperm were orientated correctly by the fluidic system pressure^13^. Further improvements of the nozzle led to an increased analytic capacity exceeding 20,000 sperm/s and sorting up to 6,000 of each X and Y bearing sperm with 90% accuracy^14^. Reduction in fluidic pressure from 50 to 40 psi resulted in an increased number of recoverable viable sperm^15^. The addition of further photodetectors, (at the angles of 45° and 135° relative to the detector at 0°) enabled to measure diagonally orientated sperm^16^. For improving the accuracy of determining the X and Y bearing sperm gas-based argon ion lasers were replaced by diode-pumped solid-state systems^16^. Moreover, alternative gating systems were implemented resulting in 98% female calves (published in patent US7371317B2, 2008). The most recent improvement was the development of SexedULTRA™ technology which minimizes stress on spermatozoa due to the sorting fluctuations in pH, tonicity, and temperature^17,18^. Moreover, sperm sorted using SexedULTRA™ were recently packaged in doses of 4 × 10^6^ sperm per insemination as compared to 2 × 10^6^ sperm per insemination which was used previously^19^.

Overall, the sorting process, which includes mechanical stress, staining with a fluorescent dye and increased handling time, is associated with molecular alterations in sex-sorted bovine sperm. Thus, bull sperm reveal decreased motility and longevity after sorting as well as decreased amounts of acrosome-intact sperm, reduced stability of the plasma membrane, mitochondrial damage, and impaired sperm-oviduct interactions^20–23^. In regard to in vitro embryo production (IVP) the use of sex-sorted bovine sperm has been reported to decrease in vitro embryo production yields^24–30^, and to cause aberrant embryo development^31^ and phenotypic alterations of calves born^32,33^.

To date, numerous field studies have been published regarding the reproductive outcome when using sex-sorted bovine sperm for artificial insemination (AI). The results of these studies are inconclusive. Conception rates using sex-sorted sperm for AI have either been reported to be similar^34,35^, or significantly reduced in heifers^9,10,36,37^ and lactating cows^18,19,38–41^ when using sex-sorted sperm for AI. Further to that, the effects of refinements of the sex-sorting technology such as the introduction of SexedULTRA™^17,18,35^, have not been fully elucidated. Thus, this study is the first to perform a comprehensive analysis of the reproductive performance of bovine sex-sorted sperm, covering all studies performed from the beginning of the commercialization until to date, spanning from 1999 to 2021 (22 years). For this aim we set out to perform a meta-analysis on NRR 24/60, conception rate, pregnancy rate, and calving rate as well as on the number of abortions and stillbirths in heifers and cows inseminated with sex-sorted sperm compared to conventional sperm.

## Material and methods

### Data sources and search strategy

A systemic search of the literature was conducted using Scopus (1999–2021) with the following databases included in the search: Web of Science Core Collection, BIOSIS Citation Index, BIOSIS Previews, Current Contents Connect, Derwent Innovations Index, KCI-Korean Journal Database, MEDLINE (including PubMed), Russian Science Citation Index, SciELO Citation Index. The following search strategy was implemented into Scopus: “cow” OR “cattle” OR “heifer” OR “heifers” OR “bull” OR “bovine” AND “sex-sorted” OR “sexed” OR “sexing” AND “sperm”. The reference lists of relevant articles were also searched for eligible studies that were absent from the electronic search. The systematic review and meta-analysis checklist PRISMA were used for this meta-analysis^42^. A total of 3136 results were retrieved following the search. Duplicate studies were excluded leaving a total number of 860 studies.

### Inclusion and exclusion criteria

Articles and reviews were included in the meta-analysis if: (1) The research was conducted in a species of domestic cattle (*Bos taurus* or *Bos indicus*). (2) The study conducted the analyses of fertility in vivo. Investigations utilising IVF and embryo transfer were excluded. (3) The sex sorting of sperm was accomplished via flow cytometry. Investigations utilising sexed sperm generated using Percoll density gradient centrifugation or the swim-up procedure were excluded. (4) Cows/heifers were artificially inseminated with sex-sorted sperm and compared to cows/heifers inseminated with conventional sperm. (5) The semen of more than one bull was used for the study. (6) The study numerically compared the reproductive performance of conventional (non-sorted sperm) and sex-sorted sperm (sperm sorted into discreet X or Y populations by flow cytometry) in at least one of the fertility measures of interest. The fertility measures of interest included non-return rates 24/60 (the proportion of females not subsequently rebred within 24 or 60 days following insemination), pregnancy rate (the percentage of cows eligible to become pregnant in a given time frame), calving rate (the percentage of cows eligible to calve within a given time frame), rate of abortions (percentage of non-viable calves produced between 50 and 270 days gestation) and stillbirths (percentage of calves born deceased or died within 24 h after birth). Additionally, timing of pregnancy detection was also evaluated, where early detection of pregnancy referred to pregnancies confirmed before 55 days and late detection of pregnancy referred to pregnancies confirmed on day 56 or later. Following these criteria, a total of 45 studies with 72 trials were included in the meta-analysis.

### Data extraction

For each study, which passed the inclusion criteria, the following categorical information was extracted: first author’s name, year of publication, study population (breed of cattle used, type of use (dairy or beef), reproductive age (heifers or cows), herd management (insemination during natural estrus or after synchronization), as well as the amount and type of semen used for insemination (fresh or frozen, less or more than 2.5 million sex-sorted sperm per straw). The following numerical data were extracted from the 72 trials where available: number of inseminated animals, non-return rate (NRR, 24 or 60 days after insemination), pregnancy rate, calving rates, occurrence of abortions and stillbirths including the discrimination between male and female calves.

### Statistical analyses

The meta-analysis was performed by using the software Review Manager (RevMan), Version 5.4.1, The Cochrane Collaboration 2020. MedCalc® Statistical Software version 19.6.4 (MedCalc Software Ltd, Ostend, Belgium; https://www.medcalc.org; 2021) was also used. For additional statistical analyses IBM SPSS 26.0 was used. In a first step, the data were checked for publication bias by analysing the asymmetry of funnel plots according to Sterne and Egger (2001)^43^ and by applying the Begg’s test. According to Hooijmans et al. (2014)^44^ a random-effects model was chosen because of the high heterogeneity of the trials included in the meta-analysis. Heterogeneity I^2^ was mostly more than 50% up to more than 75% which means a substantial and considerable heterogeneity, respectively^45^. For the comparison of the non-return rates (NRR24/60), pregnancy rates (PR), calving rates (CR) the impact of sex-sorting was estimated as the relative effect measures rate ratio (RR). According to Deeks and Higgins (2010)^46^ we used the DerSimonian and Laird random-effects model based on the Mantel–Haenszel methods for combining results across studies with additional weighting of each study effect. For comparison of the abortion rate and stillbirth rate, the odds ratio (OR) was calculated. The reliability of the effective measures was described by the 95% confidence interval (CI 95). In order to estimate the between-study variance tau^2^ was calculated^47,48^. The significance of the effective measures was tested using the Cochran–Mantel–Haenszel test (CMH). To quantify the heterogeneity of the effective measures, the I^2^ Index was calculated and tested for significance using the chi-square test. In view of the large heterogeneity, subgroup analyses were performed using the chi-square test. The subgroup analyses included the effects of the type of cow (dairy/beef), of the reproductive age (heifer/cow), of the type of semen (fresh/frozen), of the sperm dosage (more or less than 2.5 million sex-sorted sperm per straw), of the ULTRA sexing technology, and of herd management (insemination during natural estrus or after synchronization). Further to that, the reliability of early and late detection of pregnancy, as well as of rectal palpation and sonography, and the relationship between geographical distribution and pregnancy rate were compared by using the Cochran–Mantel–Haenszel test. The Bonferroni-Holm correction for multiple comparisons was used for pairwise comparison of the pregnancy rate ratios in geographical regions. Subgroup analyses were not performed if the total number of trials was smaller than 10. For comparison of non-return rates, pregnancy rates, calving rate, stillbirth, and abortion rates a t-test was used after the normal distribution had been confirmed by a Shapiro–Wilk test. If the data were not normally distributed, the Mann–Whitney *U* test was applied. Differences in the variance were tested for significance by using the Levene’s test. The results of the meta-analysis were visualized as forest plots. The Spearman's rank correlation (Spearman *rho*) was used to analyse the rank correlation between publication year and the effective measures. If p was < 0.05, results were considered significant.

## Results

### Systematic search, selection, and data extraction

**Figure 1:**
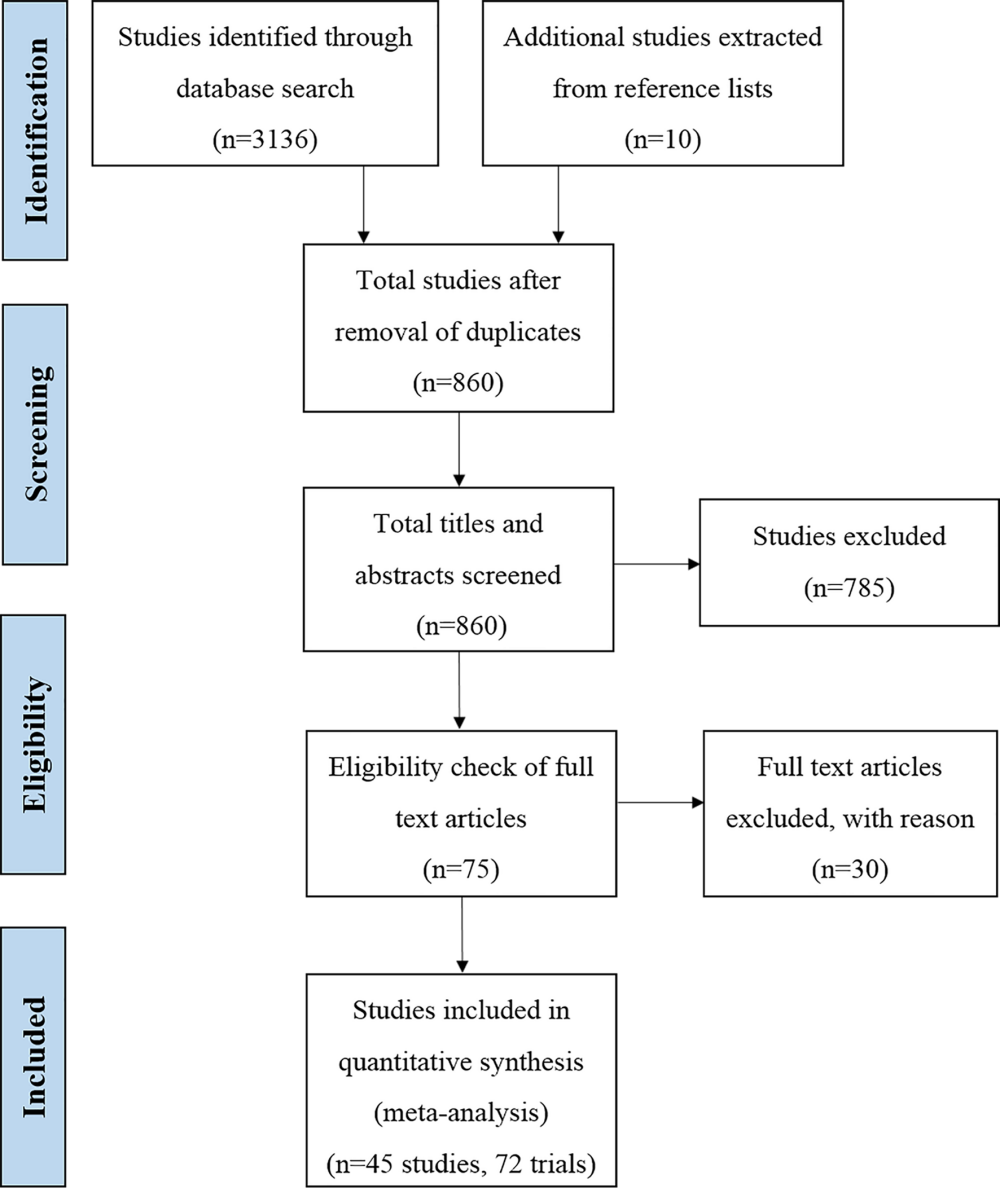
Flow diagram of search and selection strategy in the systematic review and meta-analysis of the reproductive success of bovine semen after sex-sorting.

The electronic search of Scopus returned 3,136 hits, with an additional 10 identified through the screening of reference lists. Following the removal of duplicates, 860 studies were assessed for eligibility under the inclusion criteria. After reading the titles and abstracts, 75 studies were found to directly compare conventional and sexed sperm in at least one of the fertility measures of interest. Following the inclusion criteria, 72 trials across 45 studies were eligible for inclusion in the meta-analysis (Fig. 1). Table 1 provides the descriptive data of all studies for the meta-analysis highlighting the characteristics and reproductive outcome for each trial.

**Table 1:** Descriptive data of trials included in meta-analysis. Semen: FZ: Frozen. F Fresh, US UltraSexed (frozen). ND: not determined.n_c_: number of inseminations with conventional sperm, n_s_: number of inseminations with sex-sorted sperm, Breeds: (A) Angus. (AC) Angus crossbreed. (BPL) Black Pied Lowland. (BS) Brown Swiss. (DRD) Danish Red Dairy. (H) Holstein. (HAC) Herford-Angus crossbreed. (HGC) Holstein-Gyr crossbreed. (HF) Holstein Friesian. (J) Jersey. (N) Nelore. (RA) Red Angus. (RH) Red Holstein. Management: *E* natural estrus, *S* synchronized. Outcome: PR Pregnancy rate (pregnancies/insemination), CR calving rate (births/insemination), AR Abortion rate (abortions/pregnancy), NRR Non-return rate 24–60 days after insemination, SBR Stillbirth rate (stillbirths/birth), SBR-M Stillbirth rate in male calves, SBR-F Stillbirth rate in female calves.

Publications	Population [cow/heifer (breed), inseminations management)]	Semen	Outcome (%)	
			Conventional	Sexed
1) Abdalla et al. 2014^49^	Heifers (H), n_c_ = 325, n_s_ = 426, *E*	FZ	PR 62, CR 51, AR 11	PR 34, CR 29, AR 8
2) An et al. 2010^50^	Heifers (H), n_c_ = 26, n_s_ = 36, *E*	FZ	PR 58	PR 53
3) Andersson et al. 2006^41^	Cows (HF), n_c_ = 149, n_s_ = 157, *E*	FZ	PR 46, CR 44, SBR 5	PR 21, CR 20, SBR 6
4) Bodmer et al. 2005a^51^	Cows (BS + RH), n_c_ = 64, n_s_ = 105, *E*	FZ	PR 28, CR 25, AR 6	PR 28, CR 22, AR 17
5) Bodmer et al. 2005b^51^	Heifers (BS + RH), n_c_ = 27, n_s_ = 27, *E*	FZ	PR 59, CR 58, AR 0	PR 33, CR 30, AR 11
6) Borchersen et al. 2009a^52^	Heifers (DRD), n_c_ = 153, n_s_ = 530, *E*	FZ	NRR 76, PR 65, CR 63, SBR 5, SBR-M 12, SBR-F 0,	NRR 67, PR 60, CR 56, SBR 6, SBR-M 21, SBR-F 4
7) Borchersen et al. 2009b^52^	Heifers (H), n_c_ = 181, n_s_ = 554, *E*	FZ	NRR 74, PR 62, CR 57, SBR 16, SBR-M 20, SBR-F 12	NRR 59, PR 49, CR 46, SBR 10, SBR-M 10, SBR-F 10
8) Borchersen et al. 2009c^52^	Heifers (J), n_c_ = 165, n_s_ = 504, *E*	FZ	NRR 69, PR 54, CR 50, AR 7, SBR 2, SBR-M 2, SBR-F 2	NRR 56, PR 47, CR 42, AR 11, SBR 3, SBR-M 7, SBR-F 7
9) Chebel et al. 2010^53^	Heifers (H), n_c_ = 1028, n_s_ = 343, *S*	ND	PR 52, CR 38, AR 27, SBR 3, SBR-M 5, SBR-F 1	PR 40, CR 27, AR 34, SBR 9, SBR-M 15, SBR-F 8
10) Chebel et al. 2020^54^	Heifers (H), n_c_ = 390, n_s_ = 415, *S*	ND	PR 67, CR 57, AR 15, SBR 9, SBR-M 20, SBR-F 3	PR 45, CR 40, AR 11, SBR 5, SBR-M 6, SBR-F 0
11) Colazo et al. 2017^55^	Heifers (H), n_c_ = 107, n_s_ = 117, *S*	FZ	PR 69, CR 64, AR 7	PR 64, CR 62, AR 3
12) Cooke et al. 2014^56^	Heifers and cows (HAC), n_c_ = 454, n_s_ = 439, *S*	FZ	PR 56	PR 34
13) Crites et al. 2018^35^	Heifers and cows (ND), n_c_ = 201, n_s_ = 193, *S*	US	PR 57	PR 49
14) Dawod and Elbaz 2020^36^	Heifers (H), n_c_ = 122, n_s_ = 346, *S*	US	PR 61	PR 51
15) DeJarnette et al. 2009^57^	Heifers (H), n_c_ = 53 718, n_s_ = 39 763, *E*	FZ	PR 56, SBR-M 13, SBR-F 11	PR 45, SBR-M 21, SBR-F 9
16) DeJarnette et al. 2010a^58^	Heifers (H), n_c_ = 2 089, n_s_ = 2 089, *E*	FZ	PR 61	PR 44
17) DeJarnette et al. 2010b^58^	Cows (H), n_c_ = 1 822, n_s_ = 1 822, *E*	FZ	PR 32	PR 23
18) DeJarnette et al. 2011^59^	Heifers (H), n_c_ = 2 292, n_s_ = 2 319, *E* or *S*	FZ	PR 60	PR 38
19) Djedovic et al. 2016^60^	Heifers (BPL), n_c_ = 2 115, n_s_ = 1 205, *E*	FZ	PR 55, CR 52, SBR 7	PR 44, CR 41, SBR 8
20) Dominguez et al. 2012^61^	Heifers and cows (N), n_c_ = 325, n_s_ = 338, *S*	FZ	PR 58	PR 39
21) Drake et al. 2020^39^	Heifers and cows (HF + J), n_c_ = 722 ns = 1 442, *S*	US	PR 62	PR 51
22) Duarte et al. 2007a^62^	Heifers (N), n_c_ = 83, n_s_ = 61, *E*	FZ	PR 70	PR 67
23) Duarte et al. 2007b^62^	Heifers (N), n_c_ = 103, n_s_ = 180, *S*	FZ	PR 50	PR 46
24) Frijters et al. 2009^63^	ND, n_c_ = 64 985, n_s_ = 2 036, *ND*	ND	NRR 66	NRR 53
25) Healy et al. 2013^64^	Heifers (H), ND, *S*	ND	AR 6, SBR 12, SBR-M 14, SBR-F 9	AR 6, SBR 13, SBR-M 16, SBR-F 13
26) Holden et al. 2017^65^	Heifers and cows (ND), n_c_ = 39 366, n_s_ = 1 486, *ND*	FZ	PR 54	PR 48
27) Joezy-Shekalgorabi et al*. *2017^66^	Heifers (H), n_c_ = 2 419, n_s_ = 1 154, *E*	ND	PR 64, CR 60, AR 6, SBR 5, SBR-M 5, SBR-F 4	PR 48, CR 43, AR 11, SBR 5,SBR-M 6, SBR-F 5
28) Karakaya et al. 2014a^40^	Heifers (HF), n_c_ = 66, n_s_ = 60, *S*	FZ	PR 53	PR 42
29) Karakaya et al. 2014b^40^	Cows (HF), n_c_ = 88, n_s_ = 88, *S*	FZ	PR 32	PR 25
30) Ketchum et al. 2021^67^	Heifers (A), n_c_ = 404, n_s_ = 390, *S*	US	PR 59	PR 48
31) Klinc et al. 2007^68^	Heifers (HF), n_c_ = 24, n_s_ = 22, *E*	F	PR 67	PR 55
32) Kurykin et al. 2016^69^	Heifers (H), n_c_ = 1 493, n_s_ = 1 713, *E* or *S*	FZ	PR 52	PR 42
33) Lenz et al. 2016^34^	ND, n_c_ = 62 398, n_s_ = 1 890, *ND*	US	NRR 66	NRR 67
34) Maicas et al. 2019 SS-1M^37^	Cows (HF), n_c_ = 1 593, n_s_ = 1 299, *E*	F	PR 48	PR 38
35) Maicas et al. 2019 SS-2M^37^	Cows (HF), n_c_ = 1 593, n_s_ = 1 428, *E*	F	PR 48	PR 39
36) Maicas et al. 2019 SS-FRZ^37^	Cows (HF), n_c_ = 1 593, n_s_ = 1 173, *E*	US	PR 48	PR 41
37) Maicas et al. 2019 SS-1M^37^	Heifers (HF), n_c_ = 865, n_s_ = 811, *E*	F	PR 61	PR 54
38) Maicas et al. 2019 SS-2M^37^	Heifers (HF), n_c_ = 865, n_s_ = 726, *E*	F	PR 61	PR 53
39) Maicas et al. 2019 SS-FRZ^37^	Heifers (HF), n_c_ = 865, n_s_ = 812, *E*	US	PR 61	PR 53
40) Maicas et al. 2020^19^	Cows (ND), n_c_ = 3 666, n_s_ = 3 580, *E*	US	PR 60	PR 46
41) Mallory et al. 2013^70^	Heifers (H), n_c_ = 120, n_s_ = 120, *S*	ND	PR 68	PR 38
42) Mellado et al. 2010^71^	Heifers and cows (HGC), n_c_ = 426, n_s_ = 223, *E* or *S*	FZ	PR 38	PR 23
43) Mellado et al. 2014a^72^	Heifers (H), n_c_ = 6 816, n_s_ = 15 497, *S*	ND	PR 52	PR 42
44) Mellado et al. 2014b^72^	Cows (H), n_c_ = 28 779, n_s_ = 13 574, *S*	ND	PR 24	PR 17
45) Naniwa et al. 2017a^73^	Heifers (H), n_c_ = 219, n_s_ = 524, *ND*	ND	PR 58	PR 46
46) Naniwa et al. 2017b^73^	Cows (H), n_c_ = 65, n_s_ = 214, *ND*	ND	PR 40	PR 34
47) Noonan et al. 2016^74^	Heifers (H), n_c_ = 398, n_s_ = 379, *S*	FZ	PR 60	PR 46
48) Norman et al. 2010a^75^	Heifers (H), n_c_ = 1 171 188, n_s_ = 128 702, *ND*	ND	PR 56 SBR 10, SBR-M 11, SBR-F 10	PR 39, SBR 11, SBR-M 16, SBR-F 11
49) Norman et al. 2010b^75^	Cows (H), n_c_ = 10 784 793, n_s_ = 25 910, *ND*	ND	PR 30, SBR 4, SBR-M 4, SBR-F 4	PR 25, SBR 3, SBR-M 3, SBR-F 3
50) Sá Filho et al. 2012^76^	Cows (N), n_c_ = 245, n_s_ = 246, *S*	FZ	PR 55	PR 46
51) Sales et al. 2011a^77^	Heifers (J), n_c_ = 112, n_s_ = 102, *S*	FZ	PR 52	PR 31
52) Sales et al. 2011b^77^	Cows (N), n_c_ = 193, n_s_ = 196, *S*	FZ	PR 52	PR 42
53) Schenk et al. 2009^78^	Cows (H), n_c_ = 58, n_s_ = 57, *S*	FZ	PR 55	PR 40
54) Schenk et al. 2009^78^	Cows (H), n_c_ = 713, n_s_ = 708, *S*	FZ	PR 38	PR 25
55) Seidel et al. 1999a^79^	Heifers (H), n_c_ = 118, n_s_ = 114, *S*	FZ	PR 74, CR 69, AR 6	PR 51, CR 46, AR 9
56) Seidel et al. 1999b^79^	Heifers (HAC), n_c_ = 35, n_s_ = 86, *S*	FZ	PR 51, CR 51, AR 0	PR 40, CR 40, AR 0
57) Seidel et al. 1999c^79^	Heifers (RA), n_c_ = 30, n_s_ = 14, *S*	FZ	PR 70	PR 86
58) Seidel et al. 1999d^79^	Heifers (A), n_c_ = 28, n_s_ = 45, *S*	F	PR 54, CR 32, AR 40	PR 44, CR 42, AR 5
59) Seidel et al. 1999e^79^	Heifers (AC), n_c_ = 58, n_s_ = 51, *S*	F	PR 47, AR 11	PR 33, AR 6
60) Seidel et al. 1999f^79^	Heifers (A), n_c_ = 37, n_s_ = 35, *S*	FZ	PR 73, CR 73, AR 0	PR 51, CR 51, AR 0
61) Seidel et al. 1999g^79^	Heifers (A), n_c_ = 35, n_s_ = 43, *S*	FZ	PR 57	PR 53
62) Seidel et al. 2008a^80^	Heifers (H), n_c_ = 263, n_s_ = 288, *E*	FZ	PR 62	PR 43
63) Seidel et al. 2008b^80^	Heifers (A), n_c_ = 126, n_s_ = 123, *S*	FZ	PR 67	PR 54
64) Seidel et al. 2008c^80^	Heifers (A), n_c_ = 40, n_s_ = 38, *S*	FZ	PR 73, CR 68, AR 7	PR 47, CR 42, AR 11
65) Seidel et al. 2008d^80^	Heifers (H), n_c_ = 124, n_s_ = 121, *S*	FZ	PR 60, AR 8	PR 47, AR 7
66) Seidel et al. 2008e^80^	Cows (A), n_c_ = 21, n_s_ = 42, *S*	FZ	PR 76, CR 71, AR 6	PR 57, CR 55, AR 4
67) Seidel et al. 2008f^80^	Heifers (RA), n_c_ = 30, n_s_ = 30, *S*	FZ	PR 70	PR 80
68) Thomas et al. 2014^81^	Cows (ND), n_c_ = 429, n_s_ = 422, *S*	FZ	PR 56	PR 26
69) Thomas et al. 2017^82^	Heifers (ND), n_c_ = 218, n_s_ = 217, *S*	US	PR 60	PR 52
70) Thomas et al. 2019^18^	Cows (ND), n_c_ = 812, n_s_ = 808, *S*	US	PR 65	PR 48
71) Tubman et al. 2004^83^	Heifers and cows (A + H), n_c_ = 787, n_s_ = 1 158, *S*	FZ + F	AR 5, SBR 4	AR 4, SBR 4
72) Xu 2014^84^	Cows (HF), nc = 57 085, n_s_ = 51 712, *E*	FZ	NRR 73, CR 53	NRR 69, CR 50

### Non-return rates (NRR 24/60)

**Figure 2:**
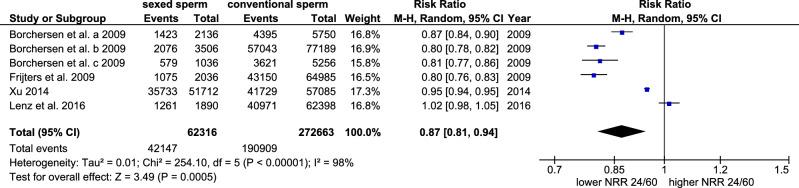
Forest plot of the non-return rates (NRR) 24/60 after the use of sex-sorted sperm compared to conventional sperm.

The NRR 24/60 was investigated in 6 trials in 3 publications (Fig. 2). The NNR 24/60 was significantly reduced from 70.7% (CI 95: 66.1–75.3 54.7–68.7) to 61.7% (CI 95: 54.7–68.7) (p < 0.001, Cochran–Mantel–Haenszel test) when using sex-sorted sperm for insemination compared to conventional sperm. The rate ratio was 0.87 (CI 95:0.81–0.94) indicating a 13% reduction in the occurrence of a successful early pregnancy 24 – 60 days after AI with sex-sorted sperm as compared to 24–60 days after AI with conventional sperm (p < 0.001, Cochran–Mantel–Haenszel test, Fig. 2). There was a statistically significant heterogeneity between trials (I^2^: 98%, tau^2^: 0.01, p < 0.001, chi-square test).

### Pregnancy rates

**Figure 3:**
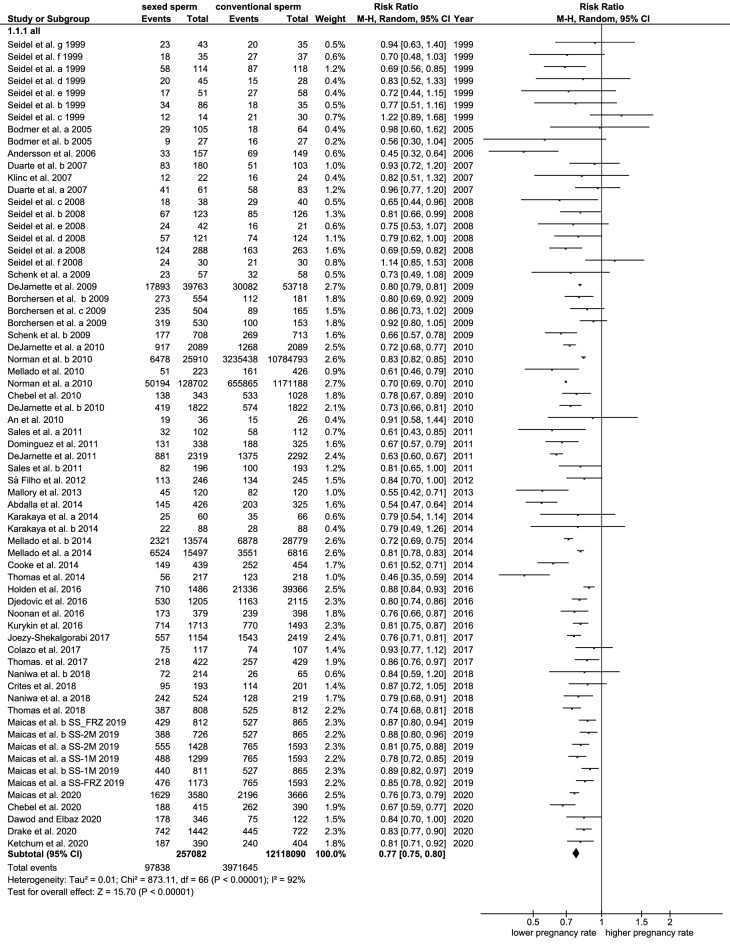
Forest plot of the pregnancy rates after the use of sex-sorted sperm compared to conventional sperm.

Pregnancy rates were investigated in 67 different trials. The overall pregnancy rates in all cows were significantly reduced from 56.1% to 43.9% when using sex-sorted sperm (p < 0.001, Cochran–Mantel–Haenszel test, Table 2). The rate ratio was 0.77 pointing to a reduction of 23% in pregnancy rates after sex-sorting (CI 95: 0.75–0.8, p < 0.001, Cochran–Mantel–Haenszel test, Table 2, Fig. 3). The heterogeneity was statistically significant (I^2^ = 93%, tau^2^ = 0.01, p < 0.001, chi-square test) confirming the use of a random-effects model and pointing to the necessity of subgroup analyses.

**Table 2:** Summary of the results of the meta-analysis of pregnancy rates after the use of sex-sorted and conventional sperm with a special focus on the effects of cow type, age, sperm freezing, sperm concentration, sperm sexing technology, timepoint of publication, and herd management. p: determination of statistical significances of: 1: the differences between two subgroups of cows inseminated with sex-sorted sperm using the t-test or Mann–Whitney *U* test. 2: the differences between two subgroups with conventional semen using the t-test or Mann–Whitney *U* test. 3: the determined rate ratio and odds ratio, respectively, using the Cochran–Mantel–Haenszel test (CMH). 4: the heterogeneity using the chi-square test. 5: the differences between subgroups inseminated with sex-sorted and conventional sperm using the CMH.

Subgroups	Trials (n)	Sexed sperm (mean, CI 95)	P^1^	conv. Sperm (mean, CI 95)	P^2^	Rate ratio	P^3^	Heterogenity I^2^/tau^2^	P^4^	P^5^ rate ratio subgroups
Total	67	43.9% (40.9–47.0)		56.1% (53.4–58.8)		0.77 (0.75–0.80	< 0.001	93%/0.01	< 0.001	
Dairy	45	41.2% (37.9–44.4)	0.009	54.0% (50.4–57.6)	0.024	0.77 (0.74–0.79)	< 0.001	94%/0.01	< 0.001	0.450
Beef	21	49.7% (43.2–56.2)		60.7% (56.7–64.6		0.79 (0.73–0.86)	< 0.001	65%/0.02	< 0.001	
Heifers	43	48.4% (45.0–51.8)	< 0.001	60.2% (58.4–62.6)	0.001	0.79 (0.75–0.82	< 0.001	93%/0.01	< 0.001	0.240
Cows	18	34.4% (28.8–39.9)		46.2% (39.3–53.2)		0.75 (0.72–0.80)	< 0.001	81%/0.01	< 0.001	
Frozen semen	49	45.1% (41.3–48.9)	0.843	57.3% (54.2–60.4)	0.393	0.77 (0.75–0.80)	< 0.001	77%/0.01	< 0.001	0.008
Fresh semen	7	45.2% (37.0–53.5)		55.0% (47.6–62.3)		0.84 (0.80–0.88)	< 0.001	12%/ < 0.01	0.340	
Sperm dosage ≤ 2.5mill. per 0.25 cc straw	44	41.4% (38.4–44.4)	< 0.001	55.2% (52.0–58.3)	0.004	0.76 (0.73–0.79)	< 0.001	79%/0.01	< 0.001	0.010
Sperm dosage > 2.5mill. per 0.25 cc straw	16	55.5% (49.1–61.8)		63.5% (59.8–67.3)		0.83 (0.78–0.88)	< 0.001	52%/0.01	0.009	
conv sexing method	54	42.9% (39.3–46.7)	0.047	55.7% (52.4–59.0)	0.590	0.76 (0.73–0.78)	< 0.001	93%/0.01	< 0.001	0.002
Ultra sexing method	13	47.9% (44.5–51.3)		57.7% (54.2–61.2)		0.82 (0.79–0.86)	< 0.001	55%/ < 0.01	0.009	
Estrus	22	44.3% (39.1–49.4)	0.919	55.7% (51.0–60.4)	0.551	0.80 (0.77–0.83)	< 0.001	74%/ < 0.01	< 0.001	0.160
Synchronisation	37	45.3% (40.8–49.7)		58.0% (54.2–61.7)		0.76 (0.73–0.80)	< 0.001	66%/0.01	< 0.001	
Before 2015	45	42.3% (38.1–46.6)	0.034	55.3% (51.5–59.1)	0.496	0.74 (0.71–0.78)	< 0.001	94%/0.01	< 0.001	< 0.001
2015 or later	22	47.2% (44.3–50.2)		57.7% (54.6–60.8)		0.81 (0.79–0.84)	< 0.001	59%/ < 0.01	< 0.001	
Early detection	34	40.6% (37.2–44.1)	0.010	53.7% (49.8–57.6)	0.031	0.75 (0.72–0.79)	< 0.001	75%/0.01	< 0.001	0.130
Late detection	24	49.5% (43.0–56.0)		60.5% (56.2–64.8)		0.80 (0.75–0.85)	< 0.001	72%/0.01	< 0.001	
Rectal palpation	13	39.8% (31.4–48.2)	0.319	50.9% (43.6% (58.2)	0.050	0.77 (0.73–0.82)	< 0.001	76%/0.01	< 0.001	0.740
Sonography	46	45.91% (42.2–49.6)		58.0% (54.9–61.1)		0.78 (0.75–0.82)	< 0.001	76%/0.01	< 0.001

When comparing dairy and beef cows, pregnancy rates were significantly higher (6.7–8.5 percentage points) in beef cows compared to dairy cows irrespective of using conventional or sex-sorted sperm (p = 0.024 and p = 0.009, respectively, t-test, Table 2). Overall, the negative effect of sex-sorting on pregnancy rates was not significantly different in dairy and beef cows (p = 0.450, Cochran–Mantel–Haenszel test, Table 2).

When comparing heifers and cows, both in heifers and cows pregnancy rates were significantly reduced (p < 0.001 and p < 0.001, respectively, Cochran–Mantel–Haenszel test) when using sex-sorted sperm. In heifers, the pregnancy rate was reduced from 60.2% (CI 95: 58.4–62.6) to 48.4% (CI 95: 45.0–51.8, Table 2). The RR was 0.79 (CI 95: 0.75–0.82) pointing to a reduction in pregnancy rates of 21% in heifers. In cows, pregnancy rates were reduced from 46.2% (CI 95: 39.3–53.2) to 34.4% (CI 95: 28.8–39.9), The RR was 0.75 (CI 95: 0.72–0.80) indicating a decrease of pregnancy rates of 25% in cows. The heterogeneity was higher in heifers than in cows (I^2^: 93%, p < 0.001 in heifers, I^2^: 81%, p < 0.001 in cows, tau^2^: 0.01, chi-square test, Table 2). The subgroup comparison between heifers and cows revealed that the negative impact of sperm sexing on pregnancy rate was similar in cows and heifers (p = 0.240, Cochran–Mantel–Haenszel test, Table 2).

The reproductive success of frozen and fresh semen was analysed in 49 and 7 different trials, respectively. Pregnancy rates after sex-sorting were significantly decreased after freezing and thawing (p = 0.008, Cochran–Mantel–Haenszel test). The rate ratio was 0.77 (CI 95: 0.75–0.80) for frozen semen and 0.84 (CI 95: 0.80–0.88) for fresh semen indicating a decrease of 7 percentage points in pregnancy rate ratios when using frozen semen (Table 2). The heterogeneity was high in frozen sperm (I^2^: 77%, tau^2^: 0.01, p < 0.001, chi-square test, Table 2) and low in fresh sperm (I^2^: 12%, tau^2^: 0.01, p = 0.340, chi-square test, Table 2).

The effects of an increased sperm dosage (mostly 4 million sperm/straw) were investigated in 16 trials whereas 44 trials used a sperm concentration of 2.5 million or less. Overall, the increase of sperm dosage resulted in significantly higher pregnancy rates both in conventional sperm (p = 0.004, t-test) and in sex-sorted sperm (p < 0.001, t-test, Table 2). The increase in pregnancy rates was 14.1 percentage points (PR: 41.4% and 55.5% for the increased concentration) in sex-sorted sperm and 8.3 percentage points in conventional sperm (PR 55.2% and 63.5%, for the increased concentration). For 2.5 million sperm per straw the RR was 0.76 (CI 95: 0.73–0.79) and for the increased concentration the RR was 0.83 (CI 95: 0.78–0.88) indicating a disproportionately higher degree of improvement of pregnancy rates by 7 percentage points (p = 0.010, Cochran–Mantel–Haenszel test) in sex-sorted sperm compared to conventional sperm when increasing sperm dosage. The use of increased sperm concentration resulted in a decrease of heterogeneity (I^2^: 79%, tau^2^: 0.01, p < 0.001 in 2.5 mill/straw and I^2^: 52%, tau^2^: 0.01, p = 0.009 in > 2.5 mill/straw sperm concentration, chi-square test, Table 2).

Regarding the method of sex-sorting the Ultra sexing technology proved to achieve a significantly higher pregnancy rate compared to the conventional sexing method (p = 0.047, Mann–Whitney *U* test). The conventional sexing technology was used in 54 trials, the Ultra sexing method was applied in 13 trials. The RR was 0.82 (CI 95: 0.79–0.86) in the Ultra sexing technology compared to 0.76 (CI 95: 0.73–0.78) in the conventional method indicating a 6 percentage points increase in pregnancy rate ratio when using this technology (p = 0.002, Cochran–Mantel–Haenszel test). Further to that the heterogeneity was higher in the conventional sexing technology (I^2^: 93%, tau^2^: 0.01, p < 0.001, chi-square test, Table 2) compared to the Ultra sexing technology (I^2^: 55%, tau^2^: 0.01, p = 0.009, chi-square test, Table 2). In line with this, the sex-sorted sperm used in the trials published in the years 2016–2020 achieved significantly higher pregnancy rates compared to the trials published between 1999 and 2015 (p = 0.034, Mann–Whitney *U* test, Table 2). Further to that, the heterogeneity was significantly higher (p = 0.003, Levene’s test) in the trials published before 2015 (I^2^: 94%, tau^2^: 0.01, p < 0.001, chi-square test, Table 2) (in 2015 no respective studies were published) compared to those published after 2015 (I^2^: 59%, tau^2^: 0.01, p < 0.001, chi-square test, Table 2). When analysing the combined effects of the use of sex-sorted sperm after freezing in heifers and after 2015 (8 trials), the overall reduction of pregnancy rates was 17.7% (CI 95: 12.3–22.8). The combination of frozen sperm sorted by the SexedUltra™ Technology in heifers (2 trials) resulted in a reduction of pregnancy rates of 13.5% as compared to AI with conventional sperm (CI 95: 7.2–19.9).

**Figure 4:**
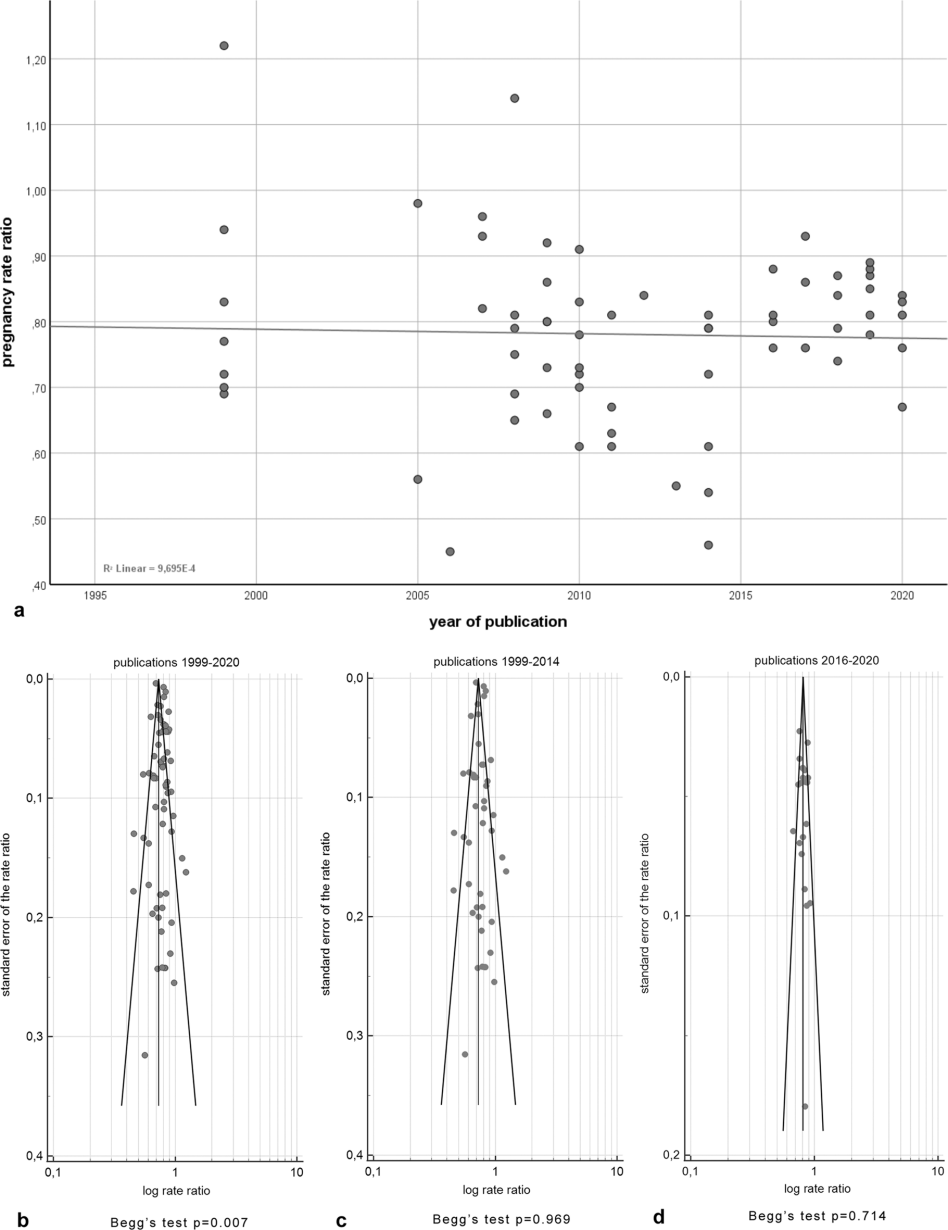
Pregnancy rate ratios in relation to the year of publication and funnel plots of studies evaluating publication bias in the reproductive success of bovine sperm after sex-sorting. (**a**) The pregnancy rate ratios show a high variation before 2015 whereas the data after 2015 reveal much less heterogeneity and an increased number of studies near the regression line. (**b**) The funnel plot of the studies published between 1999 and 2020 reveal asymmetry with outliers located besides the lines marking the 95% confidence limits. The Begg’s test reveals a significant p value of 0.007. (**c**) In the funnel plot of the studies between 1999 and 2015 there is no publication bias (p = 0.969, Begg’s test). (**d**) In the funnel plot of the publications between 2016 and 2020 the majority of values are within the 95% confidence limits and there is no publication bias (p = 0.714, Begg’s test).

When correlating the pregnancy rates with the publication year there was no correlation in the years 1999–2020 (Spearman *rho* = 0.078, p = 0.532, number of trials: 67). However, there was a correlation in the years 1999–2015 (Spearman rho = -0.305, p = 0.042, number of trials: 45). In the years 2016–2020 there was no correlation between pregnancy rate and publication year (Spearman rho = -0.085, p = 0.707, number of trials: 22). As shown in Fig. 4a, the pregnancy rate ratios show a high variation before 2015 whereas the data after 2016 reveal much less heterogeneity and an increased number of studies near the regression line (Fig. 4a).

In a last step the impact of herd management on the reproductive success was investigated. When comparing pregnancy rates after insemination during natural estrus (22 trials) or after synchronization (37 trials) pregnancy rates were not significantly different irrespective of the use of conventional or sex-sorted sperm (p = 0.551 and 0.919, respectively, Mann–Whitney *U* test, Table 2). Thus, the negative impact of sex-sorting on pregnancy rates was similar in inseminations during estrus and after synchronisation (p = 0.160, Cochran–Mantel–Haenszel test). The RR was 0.8 (CI 95: 0.77–0.83) after insemination during estrus and 0.76 (CI 95: 0.73–0.80) after synchronisation. The heterogeneity was substantial both in estrus and after synchronization (I^2^: 74% and 66%, respectively, tau^2^: 0.01, p < 0.001, chi-square test, Table 2). Regarding early and late detection of pregnancy, the late detection was significantly more reliable to detect pregnancy both after the use of conventional and sex-sorted sperm (p = 0.031 and p = 0.010, t-test, respectively, Table 2). The impact of sex-sorting on pregnancy rates was not affected by the timepoint of pregnancy detection (p = 0.130, Cochran–Mantel–Haenszel test, Table 2). When comparing rectal palpation and sonography as method for the diagnosis of pregnancy sonography was more reliable to detect pregnancy rates both after insemination with conventional and sex-sorted sperm (p = 0.050 and p = 0.319, Mann–Whitney *U* test, respectively). The impact of sperm sexing was not associated with the method of pregnancy detection (p = 0.74, Table 2). The heterogeneity was the same in both methods (I^2^: 76%, tau^2^: 0.01, p < 0.001, chi-square test, Table 2).

Further to that the effects of the geographical location of the trials on the pregnancy rate were analysed. The 67 studies were performed in 20 countries of 6 regions (Africa: 1; RR 0.84; Asia: 7, RR 0.73 (CI 95: 0.64–0.84); Australia/New Zealand: 1, RR 0.76; Europe:18, RR 0.83 (CI 95: 0.80–0.86); North America: 31, RR 0.74 (CI 95: 0.71–0.78); South America: 9, RR 0.77 (CI 95: 0.71–0.83). There were significant differences related to the geographic location (Cochran–Mantel–Haenszel test., p = 0.01). When applying pairwise comparisons with Bonferroni-Holm correction for multiple testing, Europe had a significantly higher pregnancy rates ratio compared to North America (Europe vs North America: p = 0.003, Europe vs South America: p = 0.400, Europe vs Australia p = 0.480, Europe vs Asia: p = 0.400, Europe vs Africa: p = 0.900). In Europe the pregnancy rate ratio was increased by 9 percentage points compared to North America.

In order to analyse publication bias, funnel plots were calculated for the overall time period of the meta-analysis (1999–2020) as well as for the time periods 1999–2015 and 2016–2020. The funnel plot of the studies published between 1999 and 2020 revealed asymmetry with outliers located right beside the lines marking the 95% confidence limits (Fig. 4b). In the funnel plot of the studies between 1999 and 2015 (Fig. 4c) as well as in the funnel plot of the publications between 2016 and 2020 there was no asymmetry, and most values were within the 95% confidence limits (Fig. 4d). Accordingly, the Begg’s test for analysis of publication bias in the 67 trials between 1999–2020 resulted in p = 0.007 (< 0.05 is an indication for publication bias). When analysing the trials between 1999 and 2015, p was 0.969 (number of trials: 45). In the time period between 2016 and 2020, p was 0.714 (22 trials).

### Calving rates

**Figure 5:**
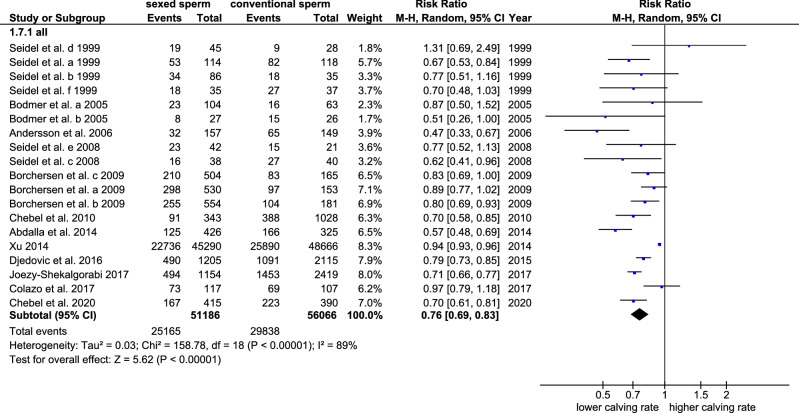
Forest plot of the calving rates after the use of sex-sorted sperm compared to conventional sperm.

Calving rates were compared in 19 trials. The overall calving rates in all cows were significantly reduced from 54.6% to 41.3% when using sex-sorted sperm (p < 0.001, Cochran–Mantel–Haenszel test, Table 3). The rate ratio was 0.76 indicating a significant decrease of 24% in calving rates after sex-sorting (CI 95: 0.69–0.83, p < 0.001, Cochran–Mantel–Haenszel test, Table 3, Fig. 5). The heterogeneity was statistically significant (I^2^ = 89%, tau^2^ = 0.03, p < 0.001, chi-square test) indicating the necessity of subgroup analyses (Table 3).

**Table 3:** Summary of the results of the meta-analysis of calving rates after the use of sex-sorted and conventional sperm with a special focus on the effects of cow type, age, sperm freezing, sperm concentration, timepoint of publication, and cow management. p: determination of statistical significances of: 1: the differences between two subgroups of cows inseminated with sex-sorted sperm using the t-test or Mann–Whitney *U* Test. 2: the differences between two subgroups with conventional semen using the t-test or Mann–Whitney *U* Test. 3: the determined rate ratio and odds ratio, respectively, using the Cochran–Mantel–Haenszel test (CMH). 4: the heterogeneity using the chi-square test. 5: the differences between subgroups inseminated with sex-sorted and conventional sperm using the CMH.

Subgroups	Trials (n)	Sexed sperm (mean, CI 95)	P^1^	conv. Sperm (mean, CI 95)	P^2^	Rate ratio	P^3^	Heterogenity I^2^/tau^2^	P^4^	p^5^ rate ratio subgroups
Total	19	41.3% (35.8–46.9)		54.6% (48.4–60.9)		0.76 (0.69–0.83)	< 0.001	89%/0.03	< 0.001	
Dairy	14	39.6.2% (32.4–46.9)	0.301	53.1% (46.4–59.7)	0.388	0.75 (0.68–0.84)	< 0.001	91%/0.03	< 0.001	0.970
Beef	5	46.0% (37.7–54.3)		59.1% (37.6–80.6)		0.76 (0.63–0.92)	0.004	0%/ < 0.01	0.430	
Heifers	15	42.5% (37.1–47.8)	0.665	56.3% (50.1–62.5)	0.469	0.75 (0.70–0.81)	< 0.001	57%/0.01	0.004	0.940
Cows	4	36.9% (8.0–65.7)		48.4% (17.9–79.0)		0.74 (0.52–1.06)	0.100	81%/0.09	0.001	
Fresh semen	2	50.2%	0.686	53.2%	0.305	0.95 (0.87–1.03)	0.220	3%/ < 0.01	0.310	< 0.001
Frozen semen	13	41.7% (33.7–49.7)		57.4% (49.4–65.4)		0.77 (0.68–0.86	< 0.001	63%/0.03	0.001	
Sperm dosage ≤ 2.5mio per 0.25 cc straw	16	40.2% (33.8–46.6)	0.209	52.9% (46.0–59.7)	0.052	0.76 (0.68–0.85)	< 0.001	89%/0.03	< 0.001	0.450
Sperm dosage > 2.5mio per 0.25 cc straw	2	50.6% (0.0–100.0		70.5% (58.2–82.8		0.69 (0.57–0.85)	0.001	0%/ < 0.01	0.550	
Before 2015	15	39.9% (33.4–46.4)	0.596	53.7% (45.7–61.6)	0.596	0.74 (0.66–0.84)-	< 0.001	82%/0.04	< 0.001	0.690
2015 or later	4	46.5% (29.6–63.5)		58.3% (49.7–66.9)		0.77 (0.69–0.85)	< 0.001	71%/0.01	0.020	
Estrus	10	37.9% (29.3–46.5)	0.183	51.4% (43.7–59.1)	0.261	0.75 (0.66–0.86)	< 0.001	93%/0.03	< 0.001	0.990
Synchronisation	9	45.1% (37.2–53.0)		58.3% (46.8–69.8)		0.75 (0.69–0.84)	< 0.001	38%/0.01	0.140

The comparison of the reproductive success in dairy (14 trials) and beef cows (5 trials) showed that the calving rates were higher (6.0–6.4 percentage points) in beef cows compared to dairy cows irrespective of using conventional or sex-sorted sperm. In view of the small and unbalanced number of trials this effect was not significant (p = 0.388 and p = 0.301, respectively, t-test, Table 3). Overall, the negative effect of sex-sorting on calving rates were similar in dairy and beef cows (p = 0.970, Cochran–Mantel–Haenszel test Table 3).

Regarding heifers and cows, calving rate ratios were not significantly different when using sex-sorted sperm (p = 0.940, Cochran–Mantel–Haenszel test, Table 3). In heifers (15 trials), the calving rate was significantly decreased from 56.3% (CI 95: 50.1–62.5) to 42.5% (CI 95: 37.1–47.8, p < 0.001, Cochran–Mantel–Haenszel test, Table 3) after sex-sorting. The RR was 0.75 (CI 95: 0.70–0.81, p < 0.001) pointing to a significant reduction in calving rates of 25% in heifers (p < 0.001, Cochran–Mantel–Haenszel test). In cows, calving rates were reduced from 48.4% (CI 95: 17.9–79.0) to 36.9% (CI 95: 8.0–65.7). The high range of the CI 95 and the lack of significance was due to the small number of 4 trials and to the high heterogeneity of the results of these trials. The RR was 0.74 (CI 95: 0.52–1.06) indicating a decrease of calving rates of 26% in cows. The heterogeneity in cows was considerably higher than in heifers (I^2^: 57%, p = 0.004 in heifers, I^2^: 81%, p = 0.001 in cows, tau^2^: 0.01, chi-square test, Table 3).

The effects of frozen and fresh semen on the calving rates were analysed in 13 and 2 trials, respectively. Calving rates after sex-sorting were significantly decreased (p < 0.001) after freezing and thawing. The rate ratio was 0.77 (CI 95: 0.68–0.86) for frozen semen and 0.95 (CI 95: 0.87–1.03) for fresh semen pointing to a reduction of 18 percentage points in calving caused by the freezing of the sex-sorted sperm (p < 0.001, Cochran–Mantel–Haenszel test Table 3). The heterogeneity was high in frozen sperm (I^2^: 63%, tau^2^: 0.03, p < 0.001, chi-square test, Table 3) whereas the heterogeneity was low in fresh sperm (I^2^: 3%, tau^2^ < 0.01, p = 0.31, chi-square test, Table 3).

The effect of a sperm dosage with more than 2.5 million sperm per straw (4 million) was only investigated in 2 trials. The effects of 2.5 million sperm or less on the calving rate were investigated in 16 trials. Overall, there was no significant impact of the sperm dose on the calving rate ratio (p = 0.450, Cochran–Mantel–Haenszel test, Table 3). For 2.5 million sperm (or less) per straw the RR was 0.76 (CI 95: 0.68–0.85) and for the increased concentration > 2.5 million sperm the RR was 0.69 (CI 95: 0.57–0.85, p < 0.001 for ≤ 2.5 million, p = 0.001 for > 2.5 million sperm per straw) (Table 3).

When comparing the effects of sperm sexing on calving rates in the trials published between 1999–2015 and in the trials published between 2016–2020 calving rates increased from 39.9% (CI 95: 33.4–46.4) to 46.5% (CI 95: 29.6–63.5) in sex-sorted sperm and from 53.7% (CI 95: 45.7–61.6) to 58.3% (CI 95: 49.7–66.9) in conventional sperm (Table 3). The RR increased from 0.74 to 0.77 (p = 0.69, Cochran–Mantel–Haenszel test, Table 3) pointing to an improvement of calving rates after sex-sorting of 3 percentage points after 2015 (Table 3). Further to that, the heterogeneity was higher in the trials published before 2015 (I^2^: 82%, tau^2^: 0.04, p < 0.001, chi-square test Table 3) compared to those published 2015 or later (I^2^: 71%, tau^2^: 0.01, p = 0.020, chi-square test, Table 3).

When correlating the calving rates with the publication year over the whole period (1999–2020) no significant relationship was found (Spearman rho =  − 0.116, p = 0.637, number of trials: 19).

In a last step the impact of herd management on calving rates was investigated. When comparing calving rates after insemination during natural estrus (10 trials) and after synchronization (9 trials) calving rates were not significantly different irrespective of the use of conventional or sex-sorted sperm (p = 0.183 and 0.261, respectively, t-test, Table 3). The negative impact of sex-sorting on calving rates was similar after inseminations during estrus or after synchronisation (p = 0.990, Cochran–Mantel–Haenszel test). Consequently, the RR was the same (0.75) after insemination during estrus and after synchronisation (CI: 0.66–0.86 and 0.69–0.84, respectively, p < 0.001, Cochran–Mantel–Haenszel test). The heterogeneity was reduced after synchronization (I^2^: 93% for insemination during estrus and 38%, for insemination after synchronisation, tau^2^: 0.03 and 0,01, respectively, p < 0.001, Table 3). Publication bias was not detected in the 19 trials analysed in the meta-analysis (Begg’s test, p = 0.087).

**Figure 6:**
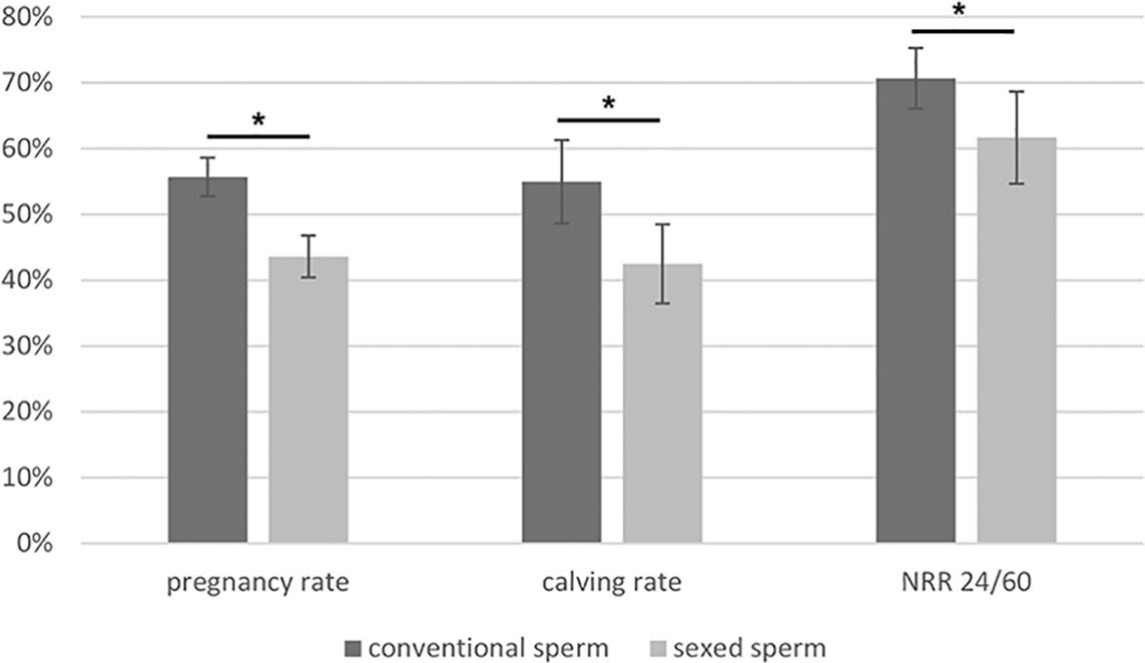
Comparison of Non-Return Rates (NRR) 24/60, pregnancy rates and calving rates after insemination with sex-sorted and conventional sperm. NRR, pregnancy rates and calving rates are significantly reduced after sex-sorting. The differences in the rates become more obvious with progression of pregnancy and reach the highest values in the calving rates. Overall, sex-sorting of sperm results in a 13% (9 percentage points) decrease of the NRR , a 23% (12.3 percentage unpoints) decrease of pregnancy rate and a 24% (13.3 percentage points) decrease of calving rate.

A summary of the impact of sperm sexing on reproductive success is provided in Fig. 6. The comparison of NRR 24/60, pregnancy rates and calving rates after insemination with sex-sorted and conventional sperm showed that all these rates were significantly reduced after sex-sorting (Fig. 6). Overall, sex-sorting of sperm resulted in a 13% (9 percentage points) decrease of the NRR, a 23% (12.3 percentage points) decrease of pregnancy rate and a 24% (13.3 percentage points) decrease of calving rate (Fig. 6).

### Abortion rates

**Figure 7:**
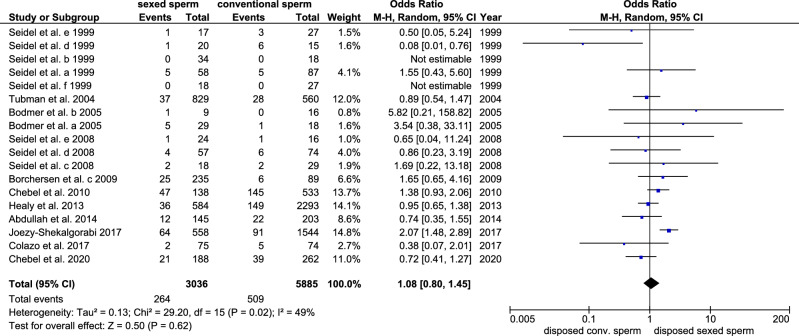
Forest plot of the abortion rates after the use of sex-sorted sperm compared to conventional sperm.

Abortion rates after insemination with conventional and sex-sorted sperm were compared in 18 trials. The abortion rates were similar when using sex-sorted and conventional sperm for insemination (8.8% (CI 95: 5.0–12.7) and 9.3% (CI 95: 4.4–14.2), respectively, p = 0.62, Cochran–Mantel–Haenszel test, Fig. 7). The odds ratio was 1.08 (CI 95: 0.8–1.45) indicating that the likelihood of the occurrence of an abortion was similar when using sex-sorted and conventional sperm. The heterogeneity was moderate (I^2^: 49%, tau^2^: 0.13, p = 0.020, chi-square test, Fig. 7). When correlating the abortion odds ratio with the publication year there was no correlation in the years 1999–2020 (Spearman rho =  − 0.010, p = 0.970). Publication bias was not present in the 18 trials analysed in the meta-analysis (Begg’s test, p = 0.787).

### Stillbirth rates

**Figure 8:**
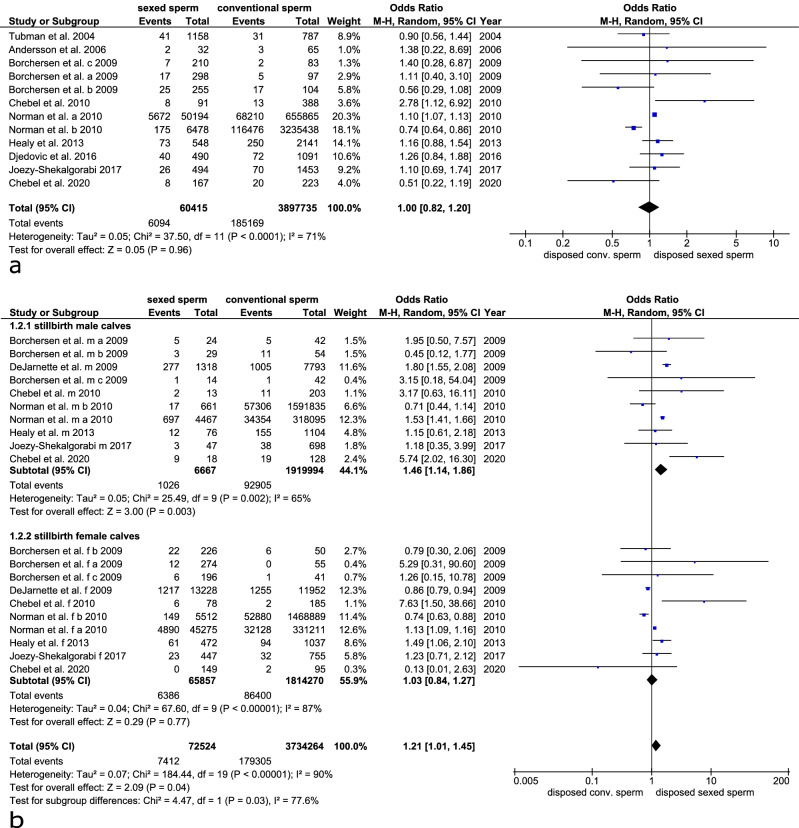
Forest plot of the stillbirth rates after the use of sex-sorted sperm compared to conventional sperm. (**a**) Forest plot including all stillbirths (**b**) Forest plot discriminating between the stillbirth of male and female calves.

Stillbirth rates were investigated in 12 trials with 10 trials discriminating between stillbirths of male and female calves. Stillbirth rates were 6.9% (CI 95: 4.7–9.1) when using sex-sorted sperm and 6.8% (CI 95: 4.2–9.5) when using conventional sperm for insemination. The odds ratio was 1.00 (CI 95: 0.82–1.20, p = 0.960, Cochran–Mantel–Haenszel test) indicating that the likelihood of the occurrence of a stillbirth was the same irrespective of using sex-sorted or conventional sperm. The heterogeneity was substantial (I^2^: 71%, tau^2^: 0.05, p < 0.001, Fig. 8a). When correlating the abortion odds ratio with the publication year there was no correlation in the years 1999–2020 (Spearman rho =  − 0.163, p = 0.612). Regarding the overall stillbirth rate publication bias was not present (Begg’s test, p = 0.784).

When discriminating between the stillbirth rates of male and female calves, the stillbirth rate of male calves was significantly increased from 10.2% (CI 95: 6.0–14.3) to 16.5% (CI 95: 7.0–26, p = 0.003, Cochran–Mantel–Haenszel test) when using sex-sorted sperm for insemination. The odds ratio for stillbirth in male calves was 1.46 (CI 95: 1.14–1,86, p = 0.003, Cochran–Mantel–Haenszel test) indicating that it is significantly more likely to experience a stillbirth in male calves after insemination with X bearing sex-sorted sperm (Fig. 8b). The stillbirth rates of female calves were similar (sex-sorted: 6.6% (CI 95: 3.6–9.5, conventional: 5.9% (2.4–9.3). The odds ratio was 1.03 (CI 95: 0.84–1.27, p = 0.77). Irrespective of the use of sex-sorted or conventional sperm the rate of stillbirths in male calves was significantly higher than in female calves (sex-sorted: p = 0.029, conventional: p = 0.043, t-test). The heterogeneity of stillbirth in female calves was higher than in male calves (female: I^2^: 87%, tau^2^: 0.04, p < 0.001, male: I^2^: 65%, tau^2^: 0.05, p = 0.002, chi-square test, Fig. 8b).

## Discussion

This meta-analysis is the first to systematically analyse the effects of bovine sex-sorting on the reproductive outcome by including publications over a time span of 22 years (1999–2021). It is also the first study to shed light on the effects of the numerous refinements of this technology to date. This knowledge is pivotal for insemination stations and farmers to be able to determine the best possible use of sex-sorted sperm and to maximize economic success. The sex-sorting technology is of high importance within the dairy industry as due to the increased number of female calves efficiencies are improved without farm expansion, thus leading to rising profit margins^4,76,85,86^. This enables dairy farmers to breed the required number of female calves and breed the remaining females to unsorted beef semen to produce high value dairy-beef cross calves. Sex-sorted sperm is also of value to the beef industry, where Y-sorted sperm may be used for AI to increase the number of male calves which represent a significant economic advantage over female calves. The fact that only between Jan 2020 and Jan 2021 32 studies on bovine sperm sexing have been published reflects the importance and economic role of sex-sorting in the cattle industry. It also emphasizes the need of the bovine industry to be able to avail of precise information on the actual reproductive success rate of bovine sperm after sex-sorting.

Our analyses showed that the overall pregnancy rates are reduced by 23% and the calving rates are diminished by 24% after sex-sorting. The finding that there is only 1% point difference between pregnancy and calving rate highlights that the major cause for the reduced reproductive outcome is mainly due to impact of sex-sorting sperm on the fertilization process and early embryonic development. The reasons for reduced fertilising capacity of sex-sorted sperm are only partly understood. During the sorting process, sperm undergo time, temperature, mechanical and chemical stress^16,34,87,88^. The analysis of a single sperm requires extensive dilution, post-sorting centrifugation to concentrate the highly diluted sexed sperm, incubation at 34–37 °C, nuclear staining with Hoechst 33342, high pressure passage through the flow cytometer, and exposure to UV laser light before collection at the base of the flow cytometer and being cooled to 5 °C^16,34,87,88^. Thus, sex-sorting results in numerous sperm alterations including reduced progressive motility^23,31^, reduced velocity^15,31^, reduced hyperactivation^31^ and abnormal movement patterns^20,31^. Additionally, reduced chromatin integrity^89^, increased ROS levels^90,91^, increased membrane permeability and reduced intracellular ATP levels^22,23,92^ as well as a shortened time to acrosome reaction have been reported^93^. Further to that, binding in the oviductal sperm reservoir is reduced^20^. Sex-sorted spermatozoa reveal deformations in the head, sharp bends in the tail and a significantly increased prevalence of damaged mitochondria^20^. These alterations are likely to be augmented by the freezing and thawing process explaining why the use of fresh sex-sorted sperm is able to achieve pregnancy rate ratios which are up to 7 percentage points higher compared to frozen sex-sorted sperm. Similarly, a significant improvement of the calving rate is seen when using fresh sex-sorted sperm. However, when interpreting this data, it has to be considered that only very few trials investigated the reproductive outcome of fresh sex-sorted sperm. Additionally, the use of fresh sex-sorted sperm is not widely applicable within the logistics of the cattle breeding industry. Interestingly, the difference in pregnancy rates was 7% points between sex-sorted and non-sorted sperm when the number of sperm per straw were increased from 2.5 million to 4 million per straw. This indicates that higher dosages of sex-sorted sperm increase the probability of the presence of an intact spermatozoon with perfect fertilizing capacity and that – within limits – the use of higher numbers of sperm is able to improve the reproductive outcome after sex-sorting. This result might also imply that some mechanical sperm defects caused by sorting may be compensable. However, it must be highlighted that only a very limited number of studies were looking at the effects of increased sperm concentrations alone. In most studies, the increased number of sperm was associated with the use of the refined SexedUltra™ technology^17–19,34^. The changes in this technology include improvements in media, reduced sorting times and incorporation of new equipment the details of which are not fully disclosed. When relating the pregnancy rates with the year of publication, it becomes clear that there is no continuous improvement of the technology and a high heterogeneity of data between 1999–2015. However, after 2015 there is rapid and highly obvious improvement in the reproductive outcome after sex-sorting. This falls in the time when SexedUltra™ technology had been developed and sperm concentrations had been increased to 4 million/straw. This rapid improvement also affects the results of the analysis of publication bias. When looking at the publication bias in all trials published between 1999 and 2020, the Begg’s test indicates publication bias when pregnancy rates were determined. However, when you look separately at the results of the trials between 1999 and 2015 and between 2016 and 2020 there is no publication bias indicating that the high heterogeneity of data and the low animal numbers before 2015 might have affected the overall result of publication bias in pregnancy rates.

The subgroup analyses confirmed that pregnancy rates are significantly higher in heifers compared to cows (sex-sorted sperm: 48% vs 34%, conventional sperm: 60% vs 46%). Interestingly there is no significant effect of sex-sorting on pregnancy rate between heifers and cows. Consequently, the preferred use of heifers for artificial insemination with sex-sorted sperm is due to the decreased fertility of the multiparous cow (reviewed in Walsh et al. 2011^94^) but is not related to the sex-sorting process itself. Similarly, the subgroup analyses showed that sex-sorting sperm impacts the field fertility of dairy and beef cattle to the same extent. Further to that the results of the analyses confirmed that beef cattle have an inherent higher fertility as opposed to dairy cattle. The long-term genetic pressure on increasing milk yields in dairy cows has caused a well-documented fertility decline^95,96^ and is being addressed in the actual dairy cattle breeding^97^. These results highlight that the alterations induced by the sex-sorting of sperm are the same in beef and dairy cows.

When comparing the different geographic localisations of the trials Europe had significantly higher pregnancy rate ratios compared to North America. This might be due to the lower herd numbers in Europe which is associated with a more intense individual observation and fertility management in Europe^98^. Additionally, the more moderate climate with lack of extreme temperatures in Europe might add to the higher pregnancy rate ratios, however escalating temperatures in northern latitudes may soon increase heat stress on animals thus reducing this advantage^99^. This finding highlights that sex-sorted sperm react in a more sensitive way to factors reducing the herd fertility than non-sorted sperm.

In regard to herd management, the meta-analysis confirmed the well-known facts that late detection of pregnancy is more reliable than early detection and that ultrasonography is superior to rectal palpation^100^. More interestingly, the reproductive outcome of sex-sorted sperm was not significantly different when the sex-sorted sperm was applied during natural estrus or after synchronization. Further studies with sex-sorted sperm are necessary to refine the distinctions between the different methods of estrus detection (observed heat, movement collar, and scratch sticker^101^) as well as the different methods of synchronisation and AI timing including split time-AI (STAI). Additional studies which explore the relationship between inherent herd fertility, herd management (including semen handling, semen placement during AI, nutrition, stabling) and reproductive performance of sex-sorted sperm for AI are paramount in achieving high pregnancy rates. In this context, it is important to note that the non-return rates 24/60 merely revealed a decrease of 13% after the use of sex-sorted sperm indicating that the NNR is overestimating reproductive success of sex-sorted sperm. This might be due to the fact that farmers either miss an unsuccessful insemination or do not get back to the insemination station after a failed insemination with sex-sorted sperm.

When looking at the overall abortion and stillbirth rates there is no significant impact of sex-sorting. However, when discriminating between male and female offspring, the stillbirth rates are 1.46 times higher for male calves after sex-sorting. This is supported by previous research which found a higher incidence of stillbirth in calves sexed for the wrong sex, i.e., in a male calf born after insemination with X-sexed semen^64,76^. Overall, this involves very few animals when considering that about 10% of the calves are the wrong sex with an extra 6 percentage points of deaths. The stillbirths may be caused by the increased formation of ROS, leading to DNA damage, mitochondrial dysfunction, and enzyme inactivation during embryonic development^102–104^. Stillbirths might also partly be due to trisomy as Y-sperm plus and extra autosome will look like an X sperm when measuring DNA content. Because of how sorts are gated, trisomies would be concentrated in semen sorted as X-sperm.

In summary, the inception of the SexedUltra™ technology in combination with increased sperm concentrations per straw represents a significant milestone in the improvement of the reproductive outcome after sex-sorting of bovine sperm. The optimal reproductive success can be achieved by applying sex-sorted sperm, which have been produced by the SexedUltra™ technology, in a concentration of 4 million sperm per straw, exclusively to heifers, which results in a reduction of pregnancy rates of 18% as compared to conventional sperm. This knowledge is pivotal for making deliberate decisions in insemination stations and individual cattle breeding farms regarding the use of sex-sorted sperm for contributing to animal welfare and for maximizing economic success. Bio economic modelling studies have shown that insemination with sex-sorted sperm in expanding herds can be used to increase farm profitability despite reduced fertility^105–107^. However, for the widespread adoption of these technology, further improvements before and after sex-sorting will have to be implemented, which mitigate the effects of sex-sorting on structure and function of the spermatozoon and support the maintenance of sperm fertilizing capacity.
